# Platelet Lysates Produced from Expired Platelet Concentrates Support Growth and Osteogenic Differentiation of Mesenchymal Stem Cells

**DOI:** 10.1371/journal.pone.0068984

**Published:** 2013-07-11

**Authors:** Sandra Mjoll Jonsdottir-Buch, Ramona Lieder, Olafur Eysteinn Sigurjonsson

**Affiliations:** 1 REModel Lab, The Blood Bank, Landspitali University Hospital, Reykjavik, Iceland; 2 Faculty of Medicine, Department of Biomedical Sciences, University of Iceland, Reykjavik, Iceland; 3 School of Science and Engineering, University of Reykjavik, Reykjavik, Iceland; Instituto Butantan, Brazil

## Abstract

**Background:**

Mesenchymal stem cells are promising candidates in regenerative cell therapy. Conventional culture methods involve the use of animal substances, specifically fetal bovine serum as growth supplement. Since the use of animal-derived products is undesirable for human applications, platelet lysates produced from human platelets are an attractive alternative. This is especially true if platelet lysates from already approved transfusion units at blood banks can be utilized. The purpose of this study was to produce human platelet lysates from expired, blood bank-approved platelet concentrates and evaluate their use as growth supplement in the culture of mesenchymal stem cells.

**Methodology/Principal Findings:**

In this study**,** bone marrow-derived mesenchymal stem cells were cultured with one of three culture supplements; fetal bovine serum, lysates from freshly prepared human platelet concentrates, or lysates from expired human platelet concentrates. The effects of these platelet-derived culture supplements on basic mesenchymal stem cell characteristics were evaluated. All cultures maintained the typical mesenchymal stem cell surface marker expression, trilineage differentiation potential, and the ability to suppress *in vitro* immune responses. However, mesenchymal stem cells supplemented with platelet lysates proliferated faster than traditionally cultured cells and increased the expression of the osteogenic marker gene *RUNX-2*; yet no difference between the use of fresh and expired platelet concentrates was observed.

**Conclusion/Significance:**

Our findings suggest that human platelet lysates produced from expired platelet concentrates can be used as an alternative to fetal bovine serum for mesenchymal stem cell culture to the same extent as lysates from fresh platelets.

## Introduction

Platelets are small anucleated structures of hematopoietic origin that contribute to hemostasis and wound healing by secreting growth factors and cytokines. They are produced by the fragmentation of megakaryocytes and released into the bloodstream, where they circulate for 7–10 days before being replaced [1]. Keeping the physiological levels of platelets well balanced is essential since quantitative and qualitative platelet disorders can be severe and difficult to treat, affecting the patient’s ability to respond to bleeding and form adequate platelet plugs [2]. Due to their importance, platelet contribution to hemostasis and biology has been thoroughly evaluated in numerous studies in the past [3], [4]. Increased understanding has shed light on their role in different biological processes apart from hemostasis and has fueled research focusing on therapeutic application by exploiting their growth factor secretion for potential clinical treatments such as wound healing [5]. Platelet-based biomaterials such as platelet gel, platelet glue and platelet rich plasma have recently been used in oral/maxillofacial surgery, treatment of chronic ulcers and orthopedics to facilitate healing and enhance bone-grafting following implantation [6], [7]. The possibility to use platelets to improve cell culture techniques has also been studied in the past few years, as well as their potential role in the design of novel cellular therapies. So far, the results have been promising [8]–[12].

Clinical application of cellular therapy and regenerative strategies are approaching a turning point, demanding increased focus on developing culture methods that are devoid of animal-derived products [13]–[15]. Common culture methods for the expansion of mesenchymal stem cells (MSC) involve the use of fetal bovine serum (FBS) to promote proliferation and survival. This poses an undesirable risk for patients in the clinical setting, as animal-derived products may cause immune-reactions towards foreign factors as well as cross-species pathogen infections [14], [16]. In the quest for replacing traditionally used FBS as growth supplement in cell culture media, recent research has focused on the application of platelet-derived products, e.g., platelet lysates that can be produced from regular platelet transfusion units by lysis [8], [11], [17]. Based on these studies, platelet-derived products have been suggested as viable alternative for the *ex vivo* culture of cells for human therapy [9], [12], [18].

Although human platelet lysates (HPL) are emerging as a possible option in the replacement of FBS in MSC cell culture, caution is advised when handling products of human origin in order to avoid transmission of infectious diseases. Therefore, it is preferable that HPL applied in the clinical setting be manufactured according to the same strict regulations as practiced in the blood processing departments of blood banks and the general hospital environment [10]. Another consideration involves the use of human donors required for the production of HPL and platelet-rich plasma. To date, there is already a shortage of blood and platelet donors and it is thus undesirable to compete with blood banks and transfusion centers for much needed freshly donated platelets [19]. Platelet units have a relatively short shelf life and as a result many donors are needed to maintain the minimum number of platelet units available for transfusion at any given time. Demand for platelets varies, making it difficult for blood banks to estimate how many units are required. It has been reported that up to 20% of all platelet units expire before they can be used and are hence discarded [20]. The shelf life of platelet units is kept short mostly because of increased risk of pathogen contamination after few days of storage at room temperature and is not related to platelet functionality [21]. After the short period of 5–7 days, units are no longer considered safe for human transfusion even though adequate platelet function may still be intact [22]. Hence, it might be possible to use platelets for other purposes than transfusion after they expire. Furthermore, the expired platelet units would have been manufactured according to the accepted standards that apply to the production of transfusion materials for human patients [23].

Human treatment involving the use of MSC is likely to be applied in the clinical setting in the near future [24], [25]. Numerous clinical trials have demonstrated the safe use of MSC and the possibility of taking advantage of their trilineage differentiation potential to aid in the regeneration of mesenchymal tissues. Special emphasis has been directed towards the use of MSC in the treatment of bone and cartilage diseases, e.g., osteogenesis imperfecta, osteoarthritis, and osteoporosis [24]–[31]. Furthermore, therapeutic approaches for the treatment of autoimmune diseases and the reduction of graft-versus-host responses have been investigated based on the ability of MSC to modulate immune responses [32]–[34].

Culture and maintenance of MSC *in vitro* is simple and cells can be isolated from a variety of tissues [35], [36]. Due to the innate ability of these cells to adhere to plastic surfaces and proliferate extensively *in vitro*, intense interest in the use of these cells in the clinical setting has arisen [9], [37], [38]. It has been shown that the expansion of MSC in growth media containing HPL produced from freshly isolated platelet units instead of the traditionally used FBS is feasible [9], [11], [18]. Therefore, the aim of this study was to demonstrate for the first time that HPL derived from expired platelet units can be used as alternative to FBS similarly to HPL produced from freshly isolated platelet units. The use of expired and fresh HPL did not affect any of the generally accepted basic characteristics of MSC, i.e., cell surface antigen expression, trilineage differentiation potential, and immunomodulatory functions. However, an increase in the proliferation potential and the expression of the osteogenic marker gene RUNX-2 was observed in HPL supplemented cultures.

## Results

### HPL does not Affect Expression of MSC Surface Markers

Expression of selected surface markers: CD29, CD45, CD73, CD90, CD105 and HLA-DR, was evaluated using flow-cytometry. MSC were cultured in media supplemented with one of three different media supplements; FBS, HPL from fresh platelet concentrates (HPLF), and HPL derived from expired platelet concentrates (HPLO) for the total of three passages prior to analysis. MSC from all cultures expressed CD29, CD73, CD90 and CD105 on their surface but not CD45 and HLA-DR (Table 1). No difference in the expression of surface markers dependent on culture conditions was observed.

**Table 1:** Surface antigen expression of MSC cultured with 10% FBS, 10% HPLF or 10% HPLO.

	FBS	HPLF	HPLO
**CD 105**	+	+	+
**CD 29**	+	+	+
**CD 45**	−	−	−
**CD 73**	+	+	+
**CD 90**	+	+	+
**HLA-DR**	−	−	−

### HPL-supplemented Media Increases Proliferation of MSC

**Figure 1:**
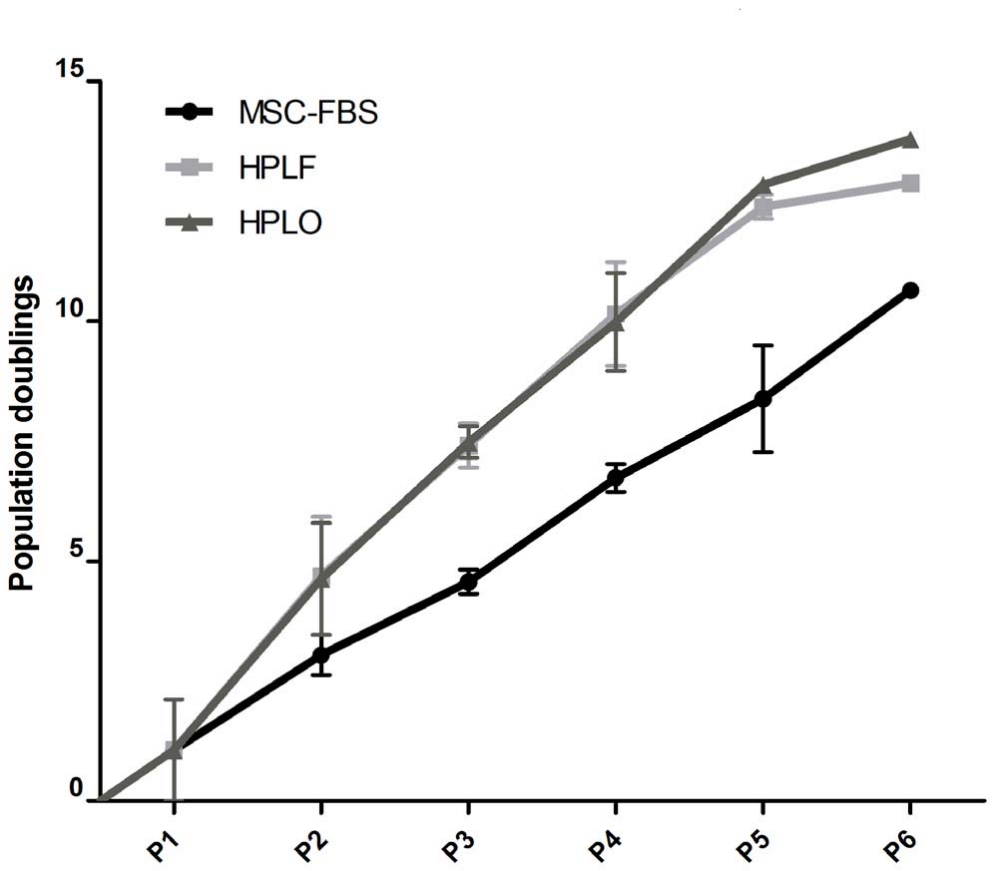
Cumulative population doublings of MSC after culture in FBS, HPLF or HPLO. MSC were cultured in culture media supplemented with 10% of FBS, platelet lysate from fresh platelet concentrates (HPLF) or lysate from expired platelet concentrates (HPLO). Population doubling assay was performed at the end of every passage for a total of six passages (P1–P6, n = 3). MSC cultured in either HPLF or HPLO consistently had higher numbers of population doublings at the end of every passage compared to MSC cultured in FBS. By the end of the sixth passage cumulative population doublings were 13.77±0.77 CPD for HPLO, 12.77±0.12 CPD for HPLF and 9.50±1.14 CPD for FBS supplemented cultures, respectively.

Cellular proliferation was evaluated by population doubling assay over six passages of culture in media containing 10% of FBS, HPLF or HPLO. Cultures supplemented with HPLF or HPLO consistently proliferated faster than FBS supplemented cultures (Figure 1). At the end of expansion over six passages, the accumulated number of total population doublings (CPD) had reached 13.77±0.77 CPD for HPLO, 12.77±0.12 CPD for HPLF and 9.50±1.14 CPD for FBS supplemented cultures, respectively. This difference was most obvious after four to five passages with a mean difference in CPD of 4.233±0.7313 (p≤0.05 ). These findings were further supported by the calculation of generation time. The average generation time between population doublings was consistently shorter in HPLF and HPLO supplemented cultures (2.27±0.20 days for HPLO, 2.71±0.37 days for HPLF and 3.41±0.13 days for FBS, respectively; p≤0.05). However, there was no significant difference in population doublings and generation time between the use of HPLF and HPLO.

### HPL Induced Morphological Changes during MSC Expansion

**Figure 2:**
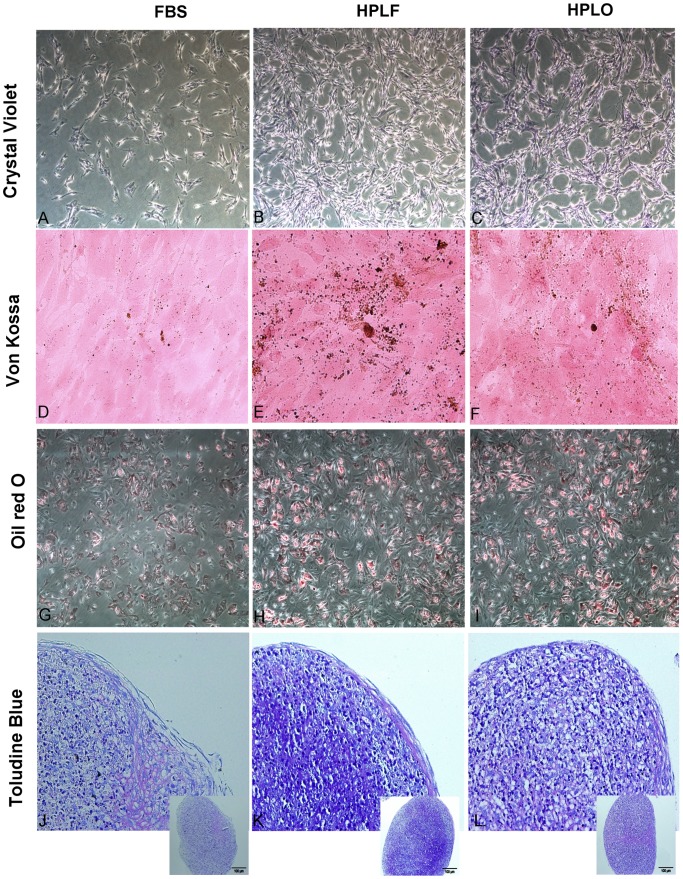
Mesenchymal stem cell morphology during expansion and differentiation. MSC were stained with crystal violet after expansion in media containing 10% of FBS, HPLF or HPLO for three passages (A–C). MSC cultured in HPLF or HPLO developed more spindle shaped morphology than cells cultured in FBS, proliferated faster and left circular areas containing no cells between them. Differentiation towards osteoblasts, adipocytes and chondrocytes was initiated after expansion in the three supplements. After 28 days of osteogenic differentiation mineralization in the culture could be visualized with a Von Kossa staining (D–F). Successful adipogenic differentiation was confirmed with Oil red O staining (G–I) and after 28 days of chondrogenic pellet cultures, the pellets were sectioned and stained with toludine blue staining (J–L). Pictures representative of three experiments.

Cell morphology was regularly evaluated during MSC culture and repeated observations revealed morphology that was dependent on the cell culture supplement. All cultures exhibited the classical fibroblast-like morphology typical of MSC, yet cells grown in HPL supplemented media were even more spindle shaped, elongated, and showed denser cell-bodies than MSC from FBS supplemented cultures (Figure 2A–C). In addition, a difference in growth behavior could be observed. MSC cultured in HPL grew in a manner that left circular areas free of cells, unlike FBS supplemented cultures.

### Trilineage Differentiation Potential Remained Unaffected by HPL

MSC in either of the three culture conditions could be successfully differentiated towards adipocytes, chondrocytes, and osteoblasts (Figure 2). After 28 days of osteogenic differentiation, mineralization was observed in all cultures, as seen by the precipitation of von Kossa stain (Figure 2D–F). Slightly more mineralization could be observed in osteogenic cultures originating from HPLF or HPLO treated MSC as compared to FBS treated. Cultures supplemented with adipogenic stimulus underwent morphological changes from a fibroblast-like appearance to round cells with distinct lipid vacuoles in the cytoplasm, which stained positive with Oil red O stain (Figure 2G–I). Chondrogenic cell-pellets from all cultures had a chondrocytic appearance with proliferating chondrocytes closer to the core of the pellet while elongated cells were observed closer to the surface, forming a sheath around the pellet in combination with extracellular matrix (figure 2J–L).

### MSCs Grown in HPL Suppress Mononuclear Cell Proliferation

**Figure 3:**
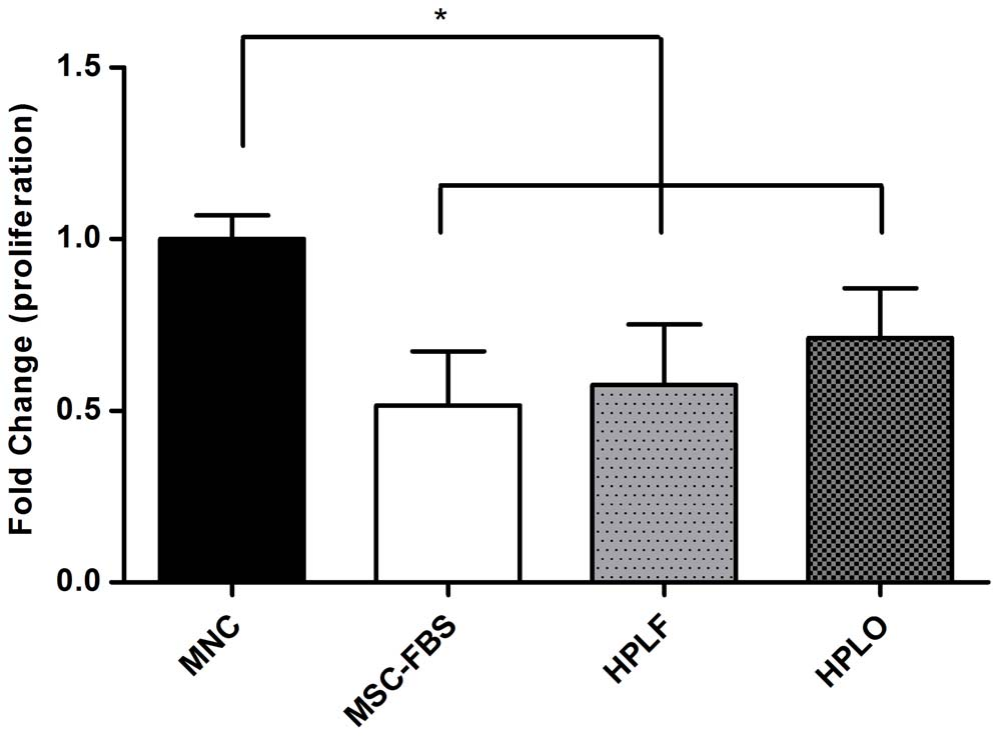
Reduction of mononuclear cell proliferation during co-culture with MSC. Mononuclear cells (MNC) were stimulated with phytohemaglutinin (PHA) and then cultured with or without MSC that had been previously expanded in media containing 10% of FBS, HPLF or HPLO. MNC proliferation was significantly less when co-cultured with MSC than if cultured on their own (p≤0.05, n = 3). The reduced proliferation was seen irrespective of the culture conditions the MSC had previously been exposed to. * = p≤0.05.

The immunomodulatory functions of MSC were evaluated by mixed lymphocyte reaction assays after cell expansion in any of the three culture supplemented media. The proliferation of stimulated mononuclear cells (MNC) co-cultured with MSC was compared to MNC proliferation in the absence of MSC. Co-culture of MSC with MNC significantly reduced the proliferation of MNC as compared to MNC alone (p≤0.05, Figure 3). The use of HPL or FBS as culture supplements during cell expansion did not affect the ability of MSC to reduce MNC proliferation.

### Alkaline Phosphatase (ALP) Activity is Temporarily Decreased in HPLF Supplemented Cultures

**Figure 4:**
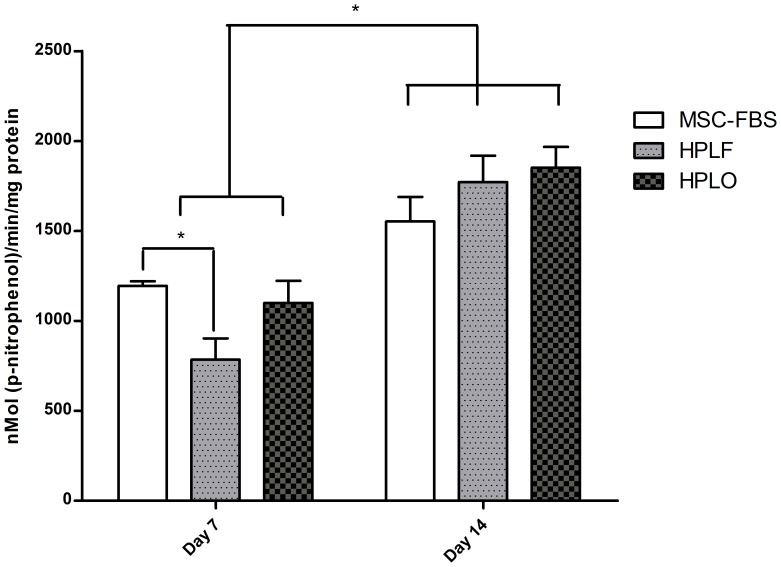
Increased enzymatic activity of alkaline phosphatase during osteogenic differentiation. MSC that had previously been cultured in media containing 10% of FBS, HPLF or HPLO underwent osteogenic differentiation. ALP activity was evaluated after 7 and 14 days of differentiation. ALP activity increased significantly from day 7 to day 14 independent of the type of culture conditions the MSC had been exposed to prior to osteogenic differentiation (p≤0.05, n = 3). * = p≤0.05.

Enzymatic activity of ALP increased during osteogenic differentiation in all MSC, independent of the supplement used. After 7 days of osteogenic differentiation, ALP activity was significantly lower in cultures supplemented with HPLF compared to FBS (1.73±0.27 nMol(p-nitrophenol)/min/mg protein for HPLF and 2.76±0.01 nMol(p-nitrophenol)/min/mg protein for FBS, p≤0.05), yet no effect could be ascribed to the use of HPLO (Figure 4). This decrease in ALP activity disappeared after 14 days in culture, removing any differences in ALP activity that could be attributed to the use of either supplement. Overall, ALP activity increased significantly from 7 to 14 days in the presence of osteogenic stimulus ranging from 1.4 fold increase for FBS and 1.86 fold increase for HPLO to 2.6 fold increase for HPLF (p≤0.05).

### Expression Pattern of Osteogenic Marker Genes

Before and during osteogenic differentiation, the expression of the three following osteogenic marker genes was analyzed, i.e., Runt –related transcription factor 2 (*RUNX2*), Secreted phosphoprotein 1 (*SPP1*), and Alkaline phosphatase (*ALP*). The choice of these specific marker genes comprehensively represents the different stages of osteogenic differentiation, with *RUNX-2* as early marker, *SPP1* as late marker, and *ALP* as steadily expressed marker gene.

**Figure 5:**
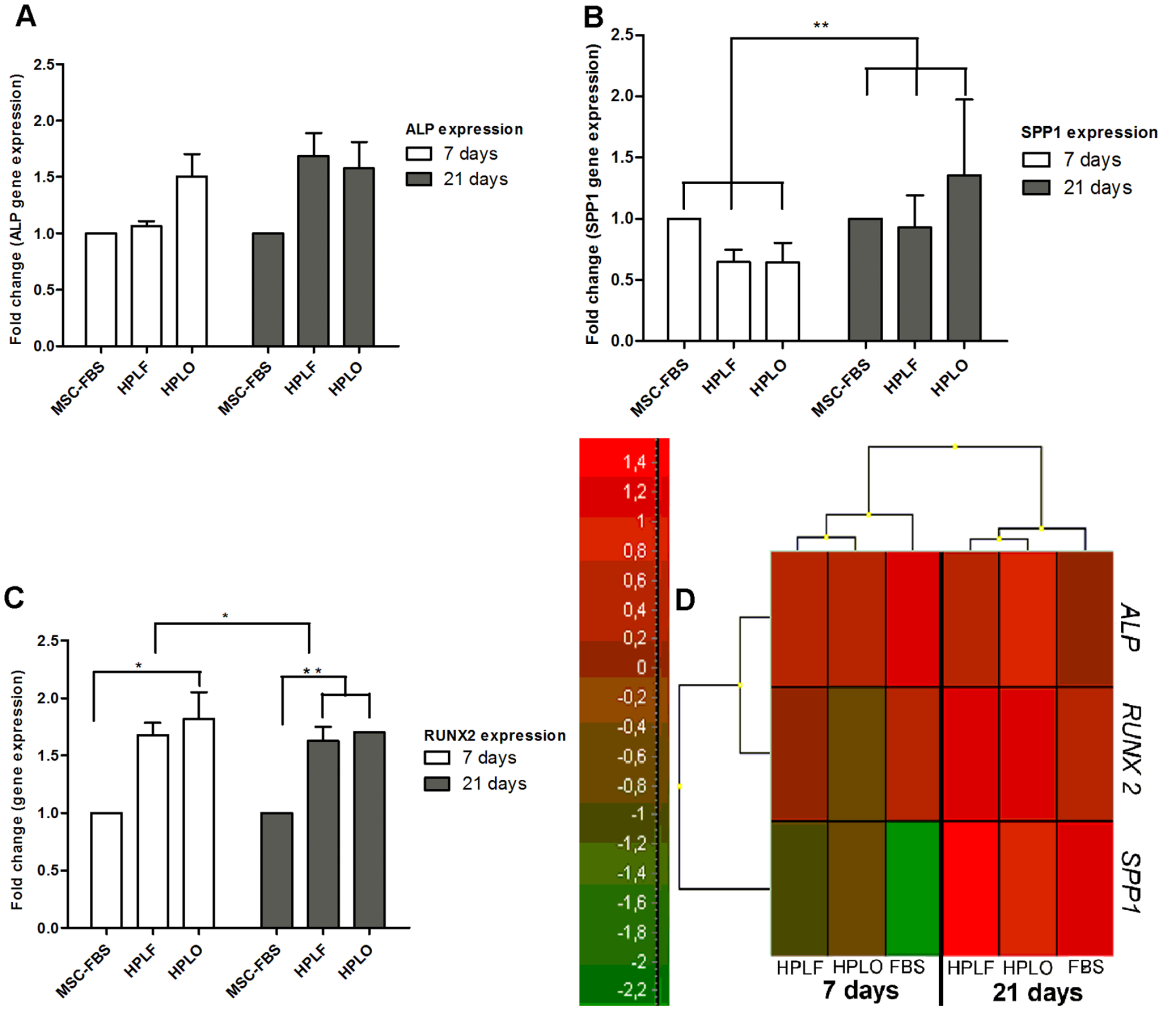
Expression of osteogenic marker genes during osteogenic differentiation of MSC. Osteogenic differentiation was initiated after MSC expansion in 10% of FBS, HPLF or HPLO. Expression of osteogenic marker genes was evaluated after 7 and 21 days of osteogenic differentiation and is represented with graphs for each gene (A–C, n = 3) and a heat-map (D) where green shades represent lower levels of expression and red shades higher levels of expression. *ALP* was evenly expressed throughout the differentiation (A,D) while the expression of *SPP1* increased from day 7 to day 21 (B,D), irrespective of the culture supplements the MSC had previously been exposed to (p≤0.01). Expression of *RUNX2* decreased from day 7 to day 21 for all cultures (C–D), still expression of *RUNX2* in cultures originating from HPLO treated MSC was higher at both time-points compared to FBS and also at day 21 for HPLF compared to FBS (p≤0.05). * = p≤0.05, ** = p≤0.01.

The expansion of MSC in FBS and HPL supplemented media did not affect the expression of osteogenic marker genes before the initiation of differentiation and thus the cells were not primed toward the osteogenic lineage during expansion. Through the course of osteogenic differentiation, *RUNX-2* expression decreased over a time period of 21 days (p≤0.01), whereas *SPP1* expression significantly increased (p≤0.01), as expected (Figure 5). Differences in *ALP* expression could be attributed to donor variation (p≤0.05), yet no effect of the culture supplements was observed.

During early time points (7 days), HPLO significantly increased the expression of *RUNX-2* (p≤0.05) as compared to HPLF and FBS supplemented cultures, which was still true after 21 days in culture (p≤0.01). No difference in *RUNX-2* expression was observed after 7 days between HPLF and FBS use, yet after 21 days in the presence of an osteogenic stimulus, HPLF, similar to HPLO, significantly increased *RUNX-2* expression as compared to FBS supplemented cultures (p≤0.01). The expression of *SPP1* and *ALP* was similar at individual time-points in any of the cultures evaluated.

## Discussion

In this study, the use of HPL prepared from expired platelet concentrates (HPLO) as growth supplement for MSC culture was compared to HPL derived from fresh platelet concentrates (HPLF) and the traditional use of FBS. Basic MSC characteristics, i.e., surface antigen expression, trilineage differentiation potential and immune-suppression, remained unaltered. However, the use of HPL as MSC growth supplement induced morphological changes, significantly increased proliferation, and resulted in elevated levels of *RUNX-2* during osteogenic differentiation.

All cultures displayed the typical MSC morphology, with long, fibroblast-like cell bodies during expansion. HPL supplementation of the growth media resulted in morphological changes, allowing growth in higher numbers per unit area. The spherical growth pattern of HPL supplemented cultures was accompanied by circular areas free of cell growth before cultures reached confluence. Similar morphological changes have been shown in other studies using HPL supplemented media for MSC expansion. This change in morphology could be due to the presence of additional factors in HPL, which are not generally found in FBS, e.g., serotonin.

Serotonin is stored in platelets and is known to affect cellular morphology of neural cells and cause vasoconstriction of endothelial cells [39]. Since there are indications that MSC reside as pericytes within the vasculature, serotonin levels could affect cellular morphology, resulting in cell contraction. Whether the morphological changes alter MSC biological functions remains speculative but there are some indications that serotonin also plays a role in bone-tissue regulation and hence effects on MSCs might be worthy to explore [40], [41].

Proliferation in the presence of HPL as growth supplement was continuously faster than traditionally cultured MSC. This trend has been described before and also encompasses other growth supplements derived from platelet units. Since the use of expired or fresh HPL did not affect the increased rate of expansion, the utilization of expired platelet units as growth supplements for MSC expansion may be advantageous. This increase in proliferation and the rate of population doublings could be due to certain growth factors present in HPL. However, since the number of MSC population doublings *in vitro* is limited to 25–50, fast cellular proliferation in HPL may cause the cells to cease expansion at earlier passages than seen in FBS supplemented cultures. Therefore it is interesting that Horn et al. showed that MSC grown in the presence of HPL may reach higher population doublings than cells grown in FBS [42], [43].

In addition, HPL supplemented media could be used to the same extent as FBS containing media for the neutralization of trypsin during passaging of cells. This indicates that platelet lysates might have similar protective abilities generally attributed to FBS, yet without providing proteolytic inhibitors.

MSC immunomodulatory functions were maintained independent of the growth supplement used for cell expansion. The MNC population used in this study is comprised of several distinct cell populations, i.e., lymphocytes, natural killer cells, and monocytes. The lymphocyte population can be further divided into subpopulations, including T-Cells (regulatory T-cells, T-helper 1 cells, and T-helper 2 cells) as well as B-cells. The immunomodulatory abilities of MSC are strongly dependent on the immune cell population, resulting in increased proliferation of T-regulatory cells and T-helper 2 cells, whereas proliferation of T-cells, B-cells, and natural killer cells is suppressed [44], [45]. The focus of the present study was on the general effect of immunomodulatory functions rather than the individual effect of MSC on each of the immune-cell populations.

In the course of osteogenic differentiation, MSC underwent the typical morphological changes from fibroblast-like cells to cells with cuboidal shape, capable of mineralization. Enzymatic activity of ALP significantly increased between 7 and 14 days of osteogenic differentiation, indicating successful transition to osteoblasts. At early time points (7 days), HPLF supplemented cultures showed significantly less ALP activity as compared to HPLO and FBS growth supplements. However, since the activity of ALP was not significantly different at 14 days, the overall effect on the quality of osteogenic differentiation is expected to be minimal. Interestingly, ALP activity was consistently similar in HPLO, yet not HPLF, and FBS supplemented cultures.

In order to exclude the possibility of lineage-specific cell priming by use of HPL as growth supplement, the expression of osteogenic marker genes was evaluated after expansion for 7 days in the absence of an osteogenic stimulus.

No induction of osteogenic marker gene expression was observed in any of the samples prior to osteogenic differentiation; indicating that MSC expansion in HPL does not induce priming towards osteogenesis and thusly expanded cells may be used for differentiation towards any of the established mesenchymal lineages. In addition, osteogenic differentiation was verified by the expression of well-known osteogenic marker genes, i.e., *RUNX-2*, *ALP*, and *SPP1*. Expression of *SPP1* increased during the course of 21 days as expected, however, a continuous and steady expression of *ALP* was observed even though the activity of the enzymatic product was seen to increase throughout the differentiation process. This might point towards posttranslational regulation of ALP. The expression of the two marker genes, *SPP1* and *ALP*, was not affected by the growth media supplements used in this study. However, the expression of the early marker *RUNX-2* was significantly higher in HPL supplemented cultures. This was especially pronounced for HPLO as growth supplements, which increased expression even at early time points, followed by HPLF at 21 days of culture.

Expression of *RUNX2* plays a central role in committing MSCs towards the osteoprogenitor lineage and subsequently upregulates expression of other factors necessary for osteogenesis such as Wnts [46]. High *RUNX2* expression is indicative of good quality osteogenic differentiation and limits the differentiation towards other lineages. The effect on *RUNX2* gene expression is not entirely surprising, since platelet lysates can contain factors that participate in osteogenesis and normal bone turn-over, such as calcitonin and ostecalcin. Therefore, HPL may be able to enhance expression of osteogenic marker genes after initiation of osteogenesis. In turn, this may lead to faster and better quality differentiation, yet does not prime cells towards the osteogenic lineage, as discussed above. These findings are in agreement with previous publications and overall indicate successful osteogenic differentiation in all samples.

Consequently, the use of HPL supplemented media may be advantageous for faster MSC expansion and improved differentiation as compared to the traditional use of FBS. Since no difference between expired and fresh HPL could be observed, expired platelet units at blood banks and transfusion centers are an attractive option for MSC culture, instead of being discarded as is the general practice today.

### Conclusion

In the present study, the effect of platelet lysates prepared from expired (HPLO) and fresh platelet (HPLF) units was compared to the traditional use of FBS in the culture of mesenchymal stem cells (MSC). By using HPLO as MSC growth supplement, high quality osteogenic differentiation as well as increased proliferation rates can be achieved without affecting basic cell characteristics, i.e., surface antigen expression, trilineage differentiation potential, and immune-suppression. Consequently, expired platelet transfusion units, currently a waste product in blood processing departments, represent a potential substitute for the use of animal-derived FBS, decreasing the risk of pathogen contamination and immune reactions to xenogenic proteins. Furthermore, the use of expired platelet units is free of ethical concerns and does not compete with the requirements of patients for platelet transfusions. Therefore, HPL derived from expired platelet units constitutes a feasible and cost-effective replacement for the use of FBS in MSC culture.

## Materials and Methods

### Preparation of Platelet Lysates

Transfusion units of platelet rich concentrates, derived from buffy coats, were obtained from the processing department at the Blood Bank, Landspitali University Hospital, Reykjavik, Iceland. Half of the units were stored immediately at −80°C (fresh units; HPLF), whereas the other half was stored for six days at room temperature in a shaking incubator until expired, and then placed in −80°C storage (expired/outdated units; HPLO). Before processing, frozen units were thawed in a 37°C water bath, resulting in platelet lysis. After 20 min centrifugation at 4975 g, supernatants were collected and filtered through a 40 µm cell strainer (BD Falcon, Franklin lakes, NJ, USA) and the pellet was subsequently discarded. Then, the filtrate was transferred to 0.45 µm Stericups (Millipore, Billerica, MA, USA) and filtered under −0.9 bar pressure, resulting in a pool of filtered lysate, which was subsequently centrifuged for 20 min at 4975 g and filtered again through a cell strainer. Aliquots were stored at −80°C. Before use as a growth supplement in cell culture media, aliquots were centrifuged at 4975 g for 10 min and only supernatants were used.

### Cell Culture

Human, bone marrow-derived MSC, negative for HIV-I, hepatitis B- and C viruses, from three donors were obtained from Lonza, Walkersville, MD, USA. After initial expansion in Standard Mesenchymal Stem Cell Basal medium (Lonza) cells were split into three separate cultures for further expansion in DMEM/F12 media (Gibco, Grand Island, NY, USA) containing 1% penicillin/streptomycin (Gibco), 4 IU/ml heparin (LEO Pharma A/S, Ballerup, Denmark), and supplemented with either 10% HPLF, 10% HPLO, or 10% MSC- approved FBS (Gibco), respectively. Cells were cultured in an incubator at 5% CO_2_, 95% humidity, and 37°C. Cells from each of the three donors were cultured independently and experiments performed in triplicates.

### Population Doubling Assay

Proliferation rate was analyzed by determining population doublings (PD) at the end of each passage for six passages using the following formula [11]:
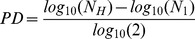
where *N_H_* denotes the number of cells retrieved at the end of the passage and *N_1_* denotes the number of cells seeded at the beginning of the passage. For the calculation of cumulative PD, the number of PD at the end of each passage was added to the PD of previous passages.

### Phenotype Confirmation

Surface antigen expression was analyzed in a FACSCalibur Flow Cytometer (Becton Dickinson, Franklin Lakes, NJ, USA) for 10^5^ cells/antibody. Antibodies were purchased from Becton Dickinson and included CD29, CD45, CD73, CD90, CD105 and HLA-DR. Cell suspensions were incubated for 20 minutes with 10 µl antibodies, diluted with PBS and vortexed prior to analysis. Data was analysed using CellQuestPro Software 4.0.2.

### Immunomodulation

The ability of MSC to suppress proliferation of mononuclear cells (MNC) stimulated with phytohemagglutinin (PHA) was analyzed using a mixed lymphocyte reaction assay (MLR). MSCs were seeded at a density of 25.000 MSC/cm^2^ in 24-well plates with RPMI 1640 media (Lonza) containing 10% MSC-FBS and allowed to adhere overnight in an incubator. The following day, media was fully replaced and semi-permeable inserts with 8 µm pores (BD Falcon) were placed in the wells and used to prevent direct contact of MSC and MNC. MNC were isolated from buffy coats of donor blood using the ficoll method [47] and seeded in RPMI 1640 medium with 10% MSC-FBS into the inserts (500.000 MNC/insert). MNC proliferation was stimulated with 1.5 µg/ml PHA (Sigma, St. Louis, MO, USA). Control wells included unstimulated MNC and stimulated MNC in the absence of MSC. MNC proliferation was analyzed after 48 h with an XTT assay following manufacturer’s instructions (ATCC, Munassas, VA, USA). Optical density was measured in a MultiSkan spectrometer (Thermo Scientific, Vantaa, Finland) at 475 nm. Measurements at 660 nm were used to reduce the signal-to-noise ratio.

### *In vitro* Differentiation

After three passages of expansion in either of the supplemented media, differentiation towards adipogenic, chondrogenic and osteogenic lineages was initiated in commercially prepared differentiation media for each lineage (Lonza, Gibco).

For osteogenic differentiation, cells were seeded in monolayers at a density of 3000 cells/cm^2^ in Mesenchymal Stem Cell Osteogenic Differentiation Media (Lonza). Mineralization was visualized with Alizarin Red Staining. Activity of alkaline phosphatase (ALP) in osteogenic cells was determined after 7 and 14 days by the conversion of p-Nitrophenolphosphate to p-Nitrophenyl (Sigma, St. Louis, MO, USA). For gene expression studies, samples were taken after 7 and 21 days of osteogenic differentiation.

Chondrocytic differentiation was performed in pellet cultures using complete Mesenchymal Stem Cell Chondrocyte Differentiation Medium (Lonza) with an initial seeding density of 250.000 cells/pellet and cultured for 28 days. For staining with toluidine blue, pellets were collected and sectioned following standard procedures.

For adipocytic differentiation, 10.000 cells/cm^2^ were seeded in monolayers with StemPro® Adipogenesis Differentiation medium (Gibco) and maintained for 14 days. Adipocytic cultures were stained with Oil red O staining for visualization of lipid vacuoles.

Images were taken in an inverted microscope using Infinity Capture 2.0 Software (Lumenerea, Capella court, Ottawa, ON, Canada).

### Gene Expression Analysis

Real-Time qPCR for the osteogenic marker genes runt-related transcription factor 2 (*RUNX2), ALP* and secreted phosphoprotein 1 *(SPP1)* was performed on samples obtained before iniation of differentiation and after 7 and 21 days in culture. RNA extraction, cDNA synthesis and Real-Time PCR was performed by TATAA Biocenter, Sweden.

Briefly, RNA was isolated using the RNeasy® Mini Kit (Qiagen, Hamburg, Germany) in a Qiagen BioRobot Workstation (Qiagen). During extraction, gDNA contamination was reduced by treating samples with RNase free DNase Set (Qiagen). Extracted RNA was analyzed for RNA quantity and quality with a NanoDrop ND-1000 spectrophotometer (Thermo Scientific). All steps were performed according to manufacturer‘s instructions.

Reverse transcription was performed with TATAA GrandScripTTM kit (TATAA Biocenter AB, Gothenburg, Sweden) in single 20 µl reactions. Reverse transcription controls were included to monitor the presence of gDNA.

RT-qPCR analysis was performed on all samples with *RUNX2*, *ALP* and *SPP1* as genes of interest. All qPCR assays were performed in duplicate in a 10 µl reaction volume using the LightCycler 480 instrument (Roche Applied Science Inc., Indianapolis, IN, USA) with TATAA SYBR® GrandMaster™ Mix (TATAA Biocenter AB). Samples were amplified for 45 cycles and analyzed with the TATAA Human Reference Gene panel. Primers for genes of interest were designed by TATAA Biocenter AB. All experiments were performed according to manufacturer’s instructions.

### Statistical Analysis

GraphPad® version 5.0 software was used for all analysis except for analysis of gene expression. GenEx 5.2.3.13 software was used to analyze gene expression. Two-way ANOVA was used where applicable and Student’s t-test was used to confirm statistical significance. P<0.05 was considered statistically significant.
